# There is more to NPH than lower body Parkinsonism

**DOI:** 10.1007/s00701-021-04904-1

**Published:** 2021-07-09

**Authors:** Joachim M. K. Oertel, Matthias J. M. Huelser

**Affiliations:** Department of Neurosurgery, Saarland University Medical Center and Saarland University Faculty of Medicine, Homburg, Saar Germany

Generally, the symptoms of normal pressure hydrocephalus (NPH) are defined by the Hakim-Trias, i.e., gait disturbance, cognitive impairment, and urinary incontinence [6]. The gait disorder of NPH is typically that of a lower body parkinsonism—a frontal gait disorder characterized as a slow, wide-based, magnetic gait pattern [20]. However, there is increasing evidence that there is a wide spectrum of different gait phenotypes in patient with NPH, ranging from unspecific to Parkinson-like [3, 16]. This condition is treatable with diversion of the cerebrospinal fluid (CSF) via shunting as the gold standard technique, with rates of improvement of up to 85% [7]. As of now, scientific evidence and clinical experience have shown that the gait disorder of NPH is the one symptom of the Hakim-Trias responding the best to shunt therapy [5, 15]. But notably, a recent study shows that the different gait patterns in NPH respond in different extents to shunt therapy [17]. Still, the improvement of gait is surely one of the main goals of shunt surgery, yet the clinical picture of NPH patients is more complex. Beside cognitive impairment and urinary incontinence, there are additional motor disturbances affecting postural stability and upper limb function, all symptoms that seem to be improved as well with shunt surgery.

The cognitive impairment of NPH patients corresponds to a subcortical-frontal dementia. Affected areas of cognition are attention, concentration, psychomotor velocity, working memory, executive functions, as well as spatial and constructive ability and flexibility of thinking [4, 14, 21]. Furthermore, the function of the temporal lobe is deteriorated, which includes verbal learning and memory function [9, 18]. But there is substantial evidence that shunt surgery significantly improves different domains of cognitive dysfunction such as verbal learning, memory, global cognitive function, and psychomotor speed [19]. Concerning others domains like the executive function a positive effect of shunting has not been proven yet [19], but there is some evidence suggesting that it might also be treatable [8, 9].

Urinary incontinence in patient with NPH is very common and occurs in up to 91% of the cases with a gender bias towards females and includes always detrusor over reactivity [10]. But, there are only a few studies investigating the characterization and the responsiveness of the different facets of the urinary incontinence in NPH patients. However, in a recent published study, the authors were able to show that shunt surgery lead to a significant improvement in urinary urgency and urge incontinence, also affecting positively the ability to perform physical activities and the overall quality of life [11].

Postural instability is part of the gait disturbance from which NPH patient suffer from. The origin of the disturbance of balance is not clear and seems to contain several different domains. There are some studies which suggest a central vestibular origin [1, 13]; others suggest a dysfunction in proprioception or in the postural center [2]. Also the postural instability can be improved after shunt surgery [13].

As above mentioned not only the lower extremity motor function is affected by the disease but also that of the upper extremities. Introduced test are the Grooved Pegboard Test [9, 22], the Finger Tapping Test [12], Line Tracing and Serial Dotting [22], and other psychomotor tests like the Trail-Testing Test [22]. Besides the scientific aspect of these findings, there could also a more practical application of such tests. For example, the Finger Tapping Test is an easily performed test with which also patients with heavily deteriorated motor function can be tested for shunt responsiveness [12]. However, generally, there are only very few studies investigating the effects of NPH to the upper motor function. The following study will fill in some gaps and without giving too much away; it introduces a further useful test for the evaluation of the upper body function; that might even be a good predictor for shunt surgery responsiveness in the future. We enjoyed reading it.

## References

[1] Abram K, Bohne S, Bublak P, Karvouniari P, Klingner CM, Witte OW, Guntinas-Lichius O, Axer H (2016). The effect of spinal tap test on different sensory modalities of postural stability in idiopathic normal pressure hydrocephalus. Dement Geriatr Cogn Dis Extra.

[2] Blomsterwall E, Svantesson U, Carlsson U, Tullberg M, Wikkelso C (2000). Postural disturbance in patients with normal pressure hydrocephalus. Acta Neurol Scand.

[3] Bugalho P, Guimaraes J (2007). Gait disturbance in normal pressure hydrocephalus: a clinical study. Parkinsonism Relat Disord.

[4] Donnet A, Schmitt A, Dufour H, Giorgi R, Grisoli F (2004) Differential patterns of cognitive impairment in patients with aqueductal stenosis and normal pressure hydrocephalus. Acta Neurochir (Wien) 146:1301–1308; discussion 1308. 10.1007/s00701-004-0384-3 10.1007/s00701-004-0384-3 15480831

[5] Grasso G, Torregrossa F, Leone L, Frisella A, Landi A (2019). Long-term efficacy of shunt therapy in idiopathic normal pressure hydrocephalus. World Neurosurg.

[6] Hakim S, Adams RD (1965). The special clinical problem of symptomatic hydrocephalus with normal cerebrospinal fluid pressure. Observations on cerebrospinal fluid hydrodynamics. J Neurol Sci.

[7] Halperin JJ, Kurlan R, Schwalb JM, Cusimano MD, Gronseth G, Gloss D (2015). Practice guideline: Idiopathic normal pressure hydrocephalus: response to shunting and predictors of response: report of the guideline development, dissemination, and implementation subcommittee of the American Academy of Neurology. Neurology.

[8] Hellstrom P, Edsbagge M, Blomsterwall E, Archer T, Tisell M, Tullberg M, Wikkelso C (2008) Neuropsychological effects of shunt treatment in idiopathic normal pressure hydrocephalus. Neurosurgery 63:527–535; discussion 535–526. 10.1227/01.NEU.0000325258.16934.BB 10.1227/01.NEU.0000325258.16934.BB 18812964

[9] Hellstrom P, Klinge P, Tans J, Wikkelso C (2012). The neuropsychology of iNPH: findings and evaluation of tests in the European multicentre study. Clin Neurol Neurosurg.

[10] Krzastek SC, Bruch WM, Robinson SP, Young HF, Klausner AP (2017). Characterization of lower urinary tract symptoms in patients with idiopathic normal pressure hydrocephalus. Neurourol Urodyn.

[11] Krzastek SC, Robinson SP, Young HF, Klausner AP (2017). Improvement in lower urinary tract symptoms across multiple domains following ventriculoperitoneal shunting for idiopathic normal pressure hydrocephalus. Neurourol Urodyn.

[12] Liouta E, Gatzonis S, Kalamatianos T, Kalyvas A, Koutsarnakis C, Liakos F, Anagnostopoulos C, Komaitis S, Giakoumettis D, Stranjalis G (2017). Finger tapping and verbal fluency post-tap test improvement in INPH: its value in differential diagnosis and shunt-treatment outcomes prognosis. Acta Neurochir (Wien).

[13] Lundin F, Ledin T, Wikkelso C, Leijon G (2013). Postural function in idiopathic normal pressure hydrocephalus before and after shunt surgery: a controlled study using computerized dynamic posturography (EquiTest). Clin Neurol Neurosurg.

[14] Mataro M, Poca MA, Del Mar MM, Catalan R, Sahuquillo J, Galard R (2003). CSF galanin and cognition after shunt surgery in normal pressure hydrocephalus. J Neurol Neurosurg Psychiatry.

[15] McGirt MJ, Woodworth G, Coon AL, Thomas G, Williams MA, Rigamonti D (2008). Diagnosis, treatment, and analysis of long-term outcomes in idiopathic normal-pressure hydrocephalus. Neurosurgery.

[16] Morel E, Armand S, Assal F, Allali G (2019). Is frontal gait a myth in normal pressure hydrocephalus?. J Neurol Sci.

[17] Morel E, Armand S, Assal F, Allali G (2021). Normal pressure hydrocephalus and CSF tap test response: the gait phenotype matters. J Neural Transm (Vienna).

[18] Ogino A, Kazui H, Miyoshi N, Hashimoto M, Ohkawa S, Tokunaga H, Ikejiri Y, Takeda M (2006). Cognitive impairment in patients with idiopathic normal pressure hydrocephalus. Dement Geriatr Cogn Disord.

[19] Peterson KA, Savulich G, Jackson D, Killikelly C, Pickard JD, Sahakian BJ (2016). The effect of shunt surgery on neuropsychological performance in normal pressure hydrocephalus: a systematic review and meta-analysis. J Neurol.

[20] Relkin N, Marmarou A, Klinge P, Bergsneider M, Black PM (2005) Diagnosing idiopathic normal-pressure hydrocephalus. Neurosurgery 57:S4–16; discussion ii-v. 10.1227/01.neu.0000168185.29659.c5 10.1227/01.neu.0000168185.29659.c5 16160425

[21] Thomas G, McGirt MJ, Woodworth G, Heidler J, Rigamonti D, Hillis AE, Williams MA (2005). Baseline neuropsychological profile and cognitive response to cerebrospinal fluid shunting for idiopathic normal pressure hydrocephalus. Dement Geriatr Cogn Disord.

[22] Tsakanikas D, Katzen H, Ravdin LD, Relkin NR (2009). Upper extremity motor measures of tap test response in normal pressure hydrocephalus. Clin Neurol Neurosurg.

